# Cooperative Gold Nanoparticle Stabilization by Acetylenic Phosphaalkenes

**DOI:** 10.1002/anie.201504834

**Published:** 2015-07-23

**Authors:** Andreas Orthaber, Henrik Löfås, Elisabet Öberg, Anton Grigoriev, Andreas Wallner, S Hassan M Jafri, Marie-Pierre Santoni, Rajeev Ahuja, Klaus Leifer, Henrik Ottosson, Sascha Ott

**Affiliations:** Department of Chemistry/Ångström Laboratories, Uppsala University Box 523, 75120 Uppsala (Sweden) E-mail: andreas.orthaber@kemi.uu.se sascha.ott@kemi.uu.se; Department of Physics and Astronomy, Uppsala University Box 516, 75120 Uppsala (Sweden); Department of Chemistry – BMC, Uppsala University Box 576, 75123 Uppsala (Sweden); Department of Engineering Sciences, Ångström Laboratories, Uppsala University Box 534, 75121 Uppsala (Sweden)

**Keywords:** ab initio studies, acetylenic phosphaalkenes, dynamic behavior, gold nanoparticles

## Abstract

Acetylenic phosphaalkenes (APAs) are used as a novel type of ligands for the stabilization of gold nanoparticles (AuNP). As demonstrated by a variety of experimental and analytical methods, both structural features of the APA, that is, the P=C as well as the C≡C units are essential for NP stabilization. The presence of intact APAs on the AuNP is demonstrated by surface-enhanced Raman spectroscopy (SERS), and first principle calculations indicate that bonding occurs most likely at defect sites on the Au surface. AuNP-bound APAs are in chemical equilibrium with free APAs in solution, leading to a dynamic behavior that can be explored for facile place-exchange reactions with other types of anchor groups such as thiols or more weakly binding phosphine ligands.

Gold clusters at the nanoscale are generally stabilized by a coordinating ligand shell.[1] Over the last years, there has been a focus on the development of novel anchoring groups for molecule–gold junctions that can overcome the insulating character of thiols which have traditionally been used in the context of molecular electronics.[2] As a general theme, these novel anchoring groups bind to the surfaces through a bond that is orthogonal to a conjugated π-system. Recent examples of this strategy include the grafting of sp-hybridized acetylene termini directly onto AuNPs or flat gold surfaces (type A, Figure 1),[3] or the direct anchoring of phenyl (sp^2^, type B) and benzyl groups (sp^3^, type C) onto Au substrates, the latter providing very efficient communication on the basis of a hyperconjugative interaction.[4]

**Figure 1:**
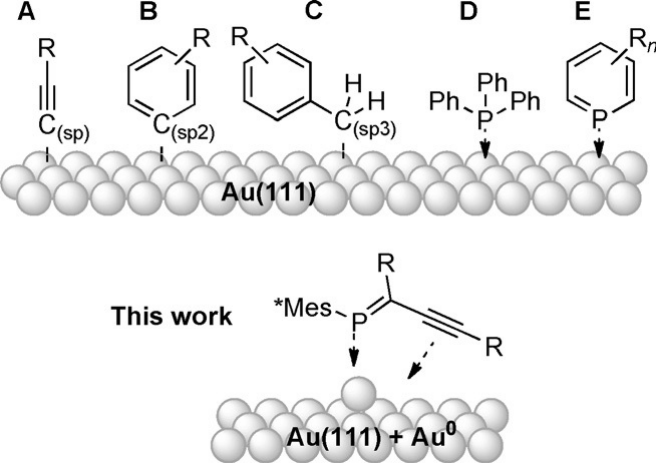
Top: Selected classes of carbon- and phosphorus-based anchor groups for gold substrates. Bottom: Cooperative binding of acetylenic phosphaalkenes in this work.

Owing to the close relationship between phosphorus and carbon,[5] also phosphorus-based ligands would most likely constitute promising candidates for this type of application, in particular as λ^3^σ^2^-phosphanes contain a lone pair for Au binding, as well as an orthogonal π-system that provides an alternative communication pathway. Stabilization and coating of defined gold clusters (e.g., eleven gold atoms)[6] and small nanoparticles (up to 1.5 nm) have hitherto been mostly limited to saturated λ^3^σ^3^-phosphanes (e.g., triphenyl phosphine, type D).[7]

To the best of our knowledge, the only example of using unsaturated phosphanes, namely λ^3^-phosphinines (type E), as ligands for AuNP stabilization is a report by Le Floch and co-workers.[8] Interestingly, these ligands induced a significant redshift of the surface plasmon resonance (SPR) compared to thiol- or phosphane-coated AuNPs,[8b] supporting the notion that sizeable communication operates across the Au–P_phosphinine_ junction. Structural integrity of the surface-bound phosphinines is however debated in view of a solid-state MAS-NMR spectroscopic study.[9]

With our interest to explore low-valent phosphorus-containing systems for molecular electronics applications,[10] we were intrigued by the possibility to use phosphaalkenes as conducting anchoring groups on Au surfaces. The inherent instability of phosphaalkenes has presumably kept many researchers from using λ^3^σ^2^-phosphanes for these kinds of purposes. This obstacle is easily met through kinetic stabilization provided by P-bound Mes* groups (Mes*=2,4,6-(*t*Bu)_3_C_6_H_2_). However, steric protection of the P=C unit could also impede strong binding of the phosphorus center to the Au surface. The present paper is the first report that shows that λ^3^σ^2^-phosphanes in the form of phosphaalkenes can stabilize AuNPs, albeit only when in conjunction with acetylenes. Experimental observations, spectroscopic data, and theoretical evidence are presented to show that both structural features of acetylenic phosphaalkenes, that is, the P=C and the C≡C units are required in a cooperative fashion for AuNP stabilization.

Initial attempts to prepare and stabilize AuNPs by the use of simple phosphaalkenes (PAs) such as **1**–**3** (Figure 2) following protocols based on various literature-known preparations of AuNPs were not met with success. For example, the reduction of HAuCl_4_ in a variety of solvents utilizing either potassium naphthalide, 9-BBN, or Et_3_SiH[11] in the presence of **1** to **3** (Figure 2) did not afford any NPs. However, the picture changes dramatically when an additional acetylene unit is introduced at the phosphaalkene moiety and one of the resulting acetylenic phosphaalkenes (APAs) **4**–**7** are employed during NP fabrication. For example, following a procedure by Wallner et al.,[11b] a mixture of HAuCl_4_ and *C*,*C*-diacetylenic phosphaalkene **7**[12] was reduced with Et_3_SiH and AuNP formation was apparent from the immediate appearance of a dark red SPR.[13] For the determination of the factors that govern NP fabrication, it was necessary to confirm that NP stabilization proceeded through the P=C unit, and not through acetylides (similar to type A, Figure 1) that could arise from desilylation of **7**. Thus, further acetylenic phosphaalkenes **4, 5**, and **6**[14] were screened for AuNP stabilization. In phosphaalkenes **5** and **6** the potentially reactive trimethylsilyl (TMS) group of **7** was replaced by robust substituents to avoid undesirable protodesilylation reactions. All phosphaalkenes that contain an additional acetylene unit in *trans* position to the Mes* group facilitate stable AuNP formation.[15]

**Figure 2:**
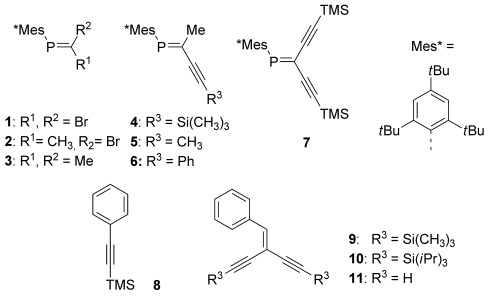
(Hetero-)Ene-yne motifs tested for AuNP preparation and stabilization. Phosphaalkenes (1–3), acetylenic phosphaalkenes (4–7), and all-carbon reference compounds (8–11).

Further control experiments to demonstrate the coordinating role of the phosphaalkenes in **4**–**7** were conducted with all-carbon-based acetylenes (**8**) and ene-diynes **9**–**11**. In all cases, the addition of reducing agents after varying mixing time did not lead to the formation of any AuNPs. These experiments clearly indicate that the P=C double bond plays a crucial role, justifying the assumption that coordination toward the AuNP surface involves the phosphorus lone pair, despite the steric demand of the Mes* group. At the same time, the experiments strongly point toward a secondary interaction between Au and the acetylene π-system.

To verify the Au-coordination to the P=C motif, molecular AuCl complexes of **1** and **7** were prepared and further tested for their suitability for AuNP preparations. Au^I^Cl complexes of **1** and **7** were prepared from [AuCl(tht)] (tht=tetrahydrothiophene).[16] X-ray crystallographic analysis (Figure 3) of two complexes shows the expected coordination of the gold atom to the phosphorus lone pair for both the acetylenic and the dibromo phosphaalkene. Both complexes exhibit short bonds ([AuCl(**7**)]: 2.206(2) and [AuCl(**1**)] 2.218(1)) compared to regular unsupported Au–P coordination (2.220(3)–2.251(2) Å).[16a,c,d]

**Figure 3:**
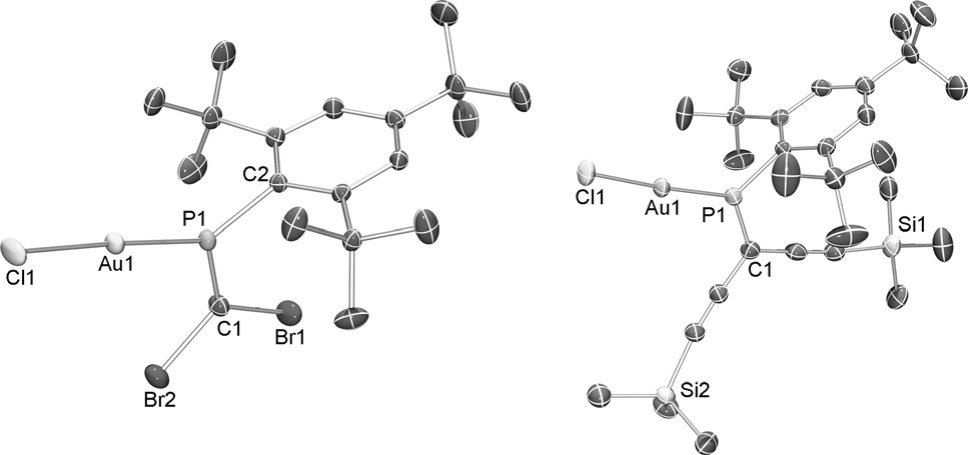
ORTEP representations of [AuCl(1)] (left) and [AuCl(7)] (right) at probability levels of 50 % and 30 %, respectively.

With [AuCl(**1**)] and [AuCl(**7**)] in hand, it was tested whether they could be reduced further to form AuNPs having already precoordinated ligands.[17] Similar to our observations with free ligands, complex [AuCl(**7**)] yielded stable AuNPs, whereas [AuCl(**1**)] was found incompatible with NP formation. Hence, it is clear that the success of the AuNP fabrication depends on the molecular structure of the stabilizing molecule, and cannot be altered by precoordination of the ligand to AuCl. The AuNPs prepared from [AuCl(**7**)] are identical to those prepared from neat **7** with respect to all characterizations described below. Noteworthy, a displacement of ligands was not observed using additional PMe_3_ or PPh_3_ ligands during the synthesis, but rather resulted in nonstabilized gold precipitates.

^1^H and ^31^P NMR spectra of the crude APA-stabilized AuNPs (see the Supporting Information, SI) only show the presence of excess free ligand, but no signals that could be attributed to ligands on the AuNP surface.[18] The absence of signals that can be assigned to surface-bound APAs is perhaps not surprising, considering their generally low concentration and the expected dramatic line broadening.[19] The APA-stabilized AuNPs could be purified by removal of all volatiles under reduced pressure, followed by extensive washing of the precipitate with cold methanol (3×50 mL). The remaining dark red solid can be quantitatively redissolved in common organic solvents such as benzene and THF without any change or loss of color. Consistent with the results from the crude reaction mixtures, NMR spectra (^1^H, ^31^P) show the absence of any detectable signals. However, and most interestingly, a significantly reduced stability of the purified AuNP solution is observed. While the crude reaction mixture, as well as the solid AuNPs, are stable over months, solutions of purified AuNPs show precipitation of elemental gold and a concomitant complete loss of color within a few hours. We attribute this behavior to a chemical equilibrium between AuNP-bound ligands and free ligands in solution. In the presence of excess ligand as is the case before purification, a sufficient amount of APAs are NP-bound to guarantee NP stabilization. Purified AuNPs are depleted of excess free ligand, and re-establishing the equilibrium leads to a significant reduction of surface-bound APAs and, concomitantly, NP decomposition.

The observed equilibrium between surface-bound ligand and free ligand in solution is an indirect proof that the molecular integrity of the APAs is maintained under the conditions for AuNP formation and that intact APA molecules are bound to the surface of the AuNP. A direct spectroscopic proof of intact ligands on the AuNP surface was obtained by surface-enhanced Raman spectroscopy (SERS). Our measurements clearly show the presence of acetylenic phosphaalkenes with bands at 2107 and 1207 cm^−1^ for the acetylene and phosphaalkene stretching vibrations, respectively (Figure 4). Both of these values are significantly shifted compared to those in the free ligand (2131 and 1130 cm^−1^), underlining the cooperative binding mode of the acetylenic phosphaalkenes.

**Figure 4:**
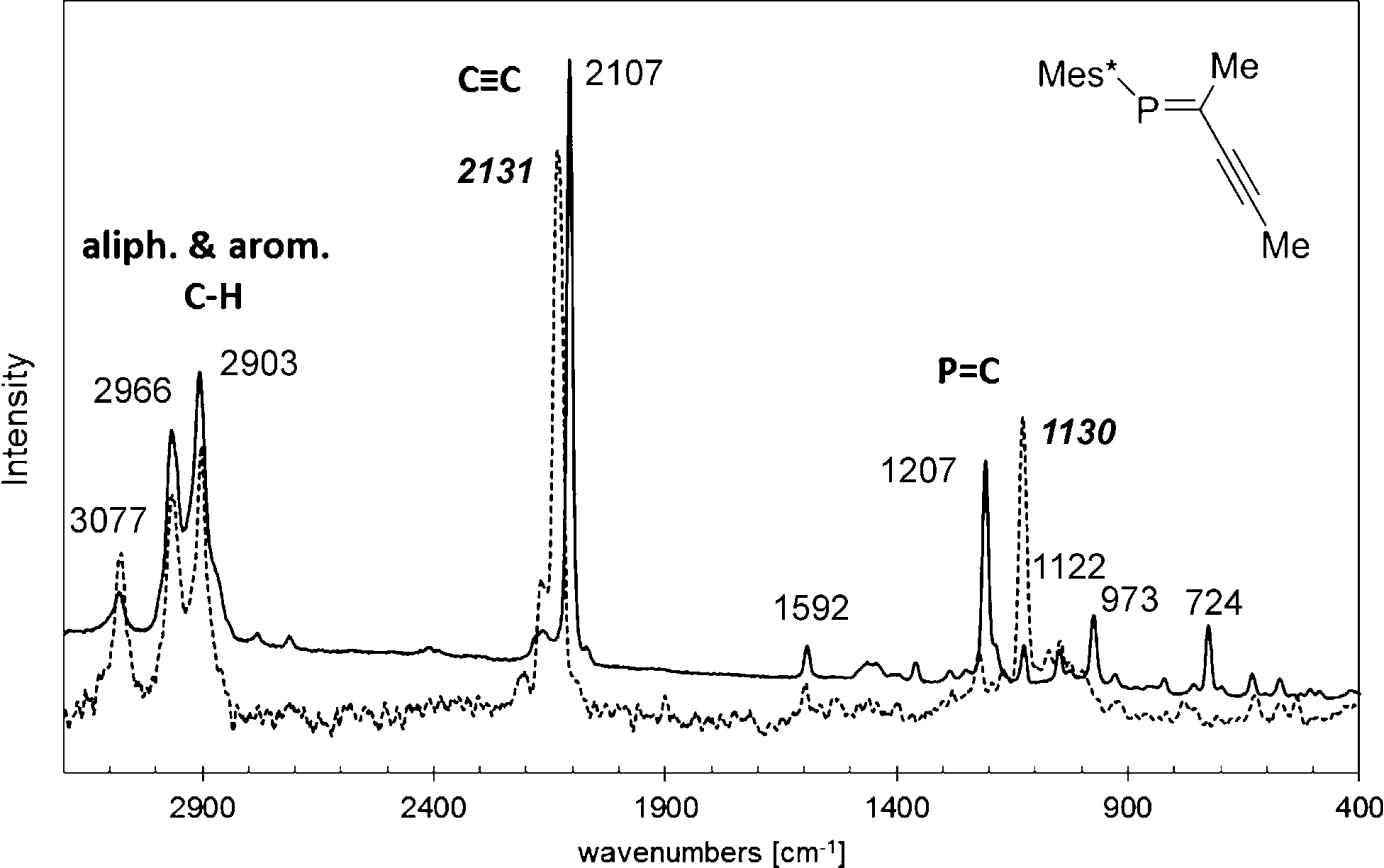
Representative Raman analysis of purified solid AuNP (solid line) in comparison with its free acetylenic phosphaalkene ligand 4 (dotted line). The shifts of the resonances associated with the acetylene and the phosphaalkene vibrations supports the cooperative binding mode that involves both structural features, that is, the P=C and the C≡C units.

Moreover, we explored the possibility to replace the labile APA ligands by means of place-exchange reactions. Hence, an excess of aryl thiol (benzene thiol or 1,2-benzene dithiol) was added to a freshly prepared AuNP[**6**] solution. Successful exchange was confirmed by means of SERS after purification of the nanoparticles. Exchange reactions with trimethyl phosphine resulted in a complete loss of color indicating that an excess of strongly binding phosphine ligands leads to NP decomposition. The decomposition of the AuNPs is initiated by a decolorization of the solution followed shortly after by the precipitation of elemental gold which indicates the rapid formation of nonstabilized gold aggregates. Interestingly, however, we were able to perform such exchange reactions with weakly binding triphenyl phosphine ligands.

The size determination by transmission electron microscopy (TEM) shows an average AuNP size of 7.9 to 9.8 nm for the different preparations (Figure 5). Irrespective of the employed APA and the preparation method, both the size and their SPR are very similar. Interestingly, we observe extensive formation of mainly Au(111) facets for these nanoparticles. However, there are also some Au(002) facets and some disorder areas found. The SPR exhibits its maximum between 520 and 530 nm, which is slightly shifted compared to similarly sized AuNPs with citrate, thiol, amine, or triphenylphopsphine ligands.[20] The observed redshift of the SPR is a first indication of interactions between the Au atoms and their coating molecules, although this interaction seems less pronounced compared to that of the strongly electron-withdrawing phosphinines (type E, Figure 1).

**Figure 5:**
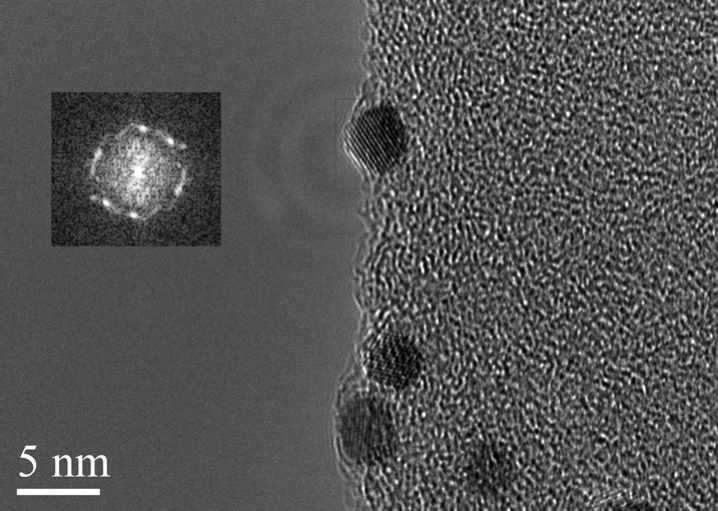
High-resolution TEM image with FFT inset showing the presence of mainly Au(111) lattice planes.

Beyond that, we were interested in understanding the surface adsorption by modeling APA **4** on gold surfaces (Table 1). We explain the coordination of phosphaalkenes by defect sites of the Au(111) lattice planes that were indicated by electron microscopy. In contrast to pristine Au(111) surface, adding a low-coordinated gold atom leads to short Au–P bonds of 2.36 Å and hence gives rise to a significant binding of ca. −24 kcal mol^−1^. The additional gold atom serves as a mediator of this interaction to the substrate, which presumably is of dative character. Direct binding of APA **4** to Au(111) is unlikely to contribute to AuNP stabilization as the Au–P bonds are significantly elongated (≈4.1 Å) and the association energies are slightly positive (ca. 0.5 kcal mol^−1^). Notably, the bond lengths within the phosphaalkene moiety do not differ significantly from those calculated for isolated compounds. As shown in Figure 6, bonding interactions of APA **4** extend into the triple bond giving rise to dominant bonding interactions between {Au,C1} (3.87 Å) and {Au,C2} (4.50 Å, for further details of this interactions see the SI).

**Figure 6:**
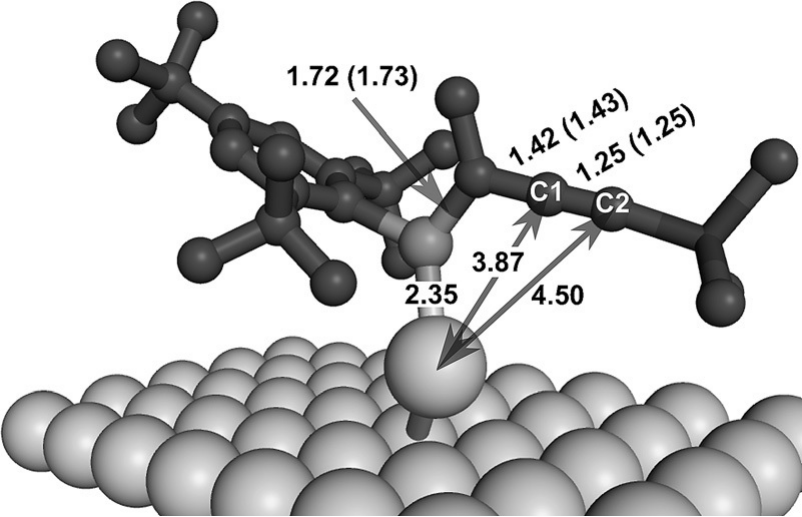
Surface-bound acetylenic phosphaalkene. Distances [Å] and angles [°] for gas-phase calculations are given in parenthesis.

**Table 1:** Calculated binding of acetylenic phosphaalkene 3 to (modified) Au(111) surface

Compound	Au–P^[c]^	P=C^[d]^	*E*_ass_^[e]^
**4**^[a]^	4.11	1.73(1.73)	0.5
**4**+adatom^[b]^	2.36	1.72(1.73)	−24.2

[a] Flat Au(111) surface. [b] Au(111) plus additional Au atom [c] Au–P distance in Å, for the flat surface the distance is defined as the distance of the P atom from the top layer of gold atoms in Au(111). [d] P=C distance in Å, values in brackets denote gas-phase distances. [e] Association energy of relaxed molecules [kcal mol^−1^].

In summary, we report the preparation of uniform facet-rich gold nanoparticles (AuNPs) with *C*-acetylenic phosphaalkene ligands in a size regime of ca. 8–10 nm. Stability arises through coordination of the phosphorus lone pair and is further enhanced by the pendant acetylenic unit. Our experimental findings are supported by surface-enhanced Raman spectroscopy (SERS) and theoretical calculations. In contrast to thiol ligands, we observe a fast dynamic behavior of APA ligands in solution. Such weakly bound ligands are highly warranted in applications that need organic solvent conditions and that require facile place exchange with other types of anchor groups such as thiols or weakly binding phosphanes. This novel class of gold nanoparticle ligands provides a facile synthesis of monodisperse AuNPs as simple precursors for subsequent exchange reactions with thiolate or weakly binding phosphane ligand systems.

## Supporting Information

As a service to our authors and readers, this journal provides supporting information supplied by the authors. Such materials are peer reviewed and may be re-organized for online delivery, but are not copy-edited or typeset. Technical support issues arising from supporting information (other than missing files) should be addressed to the authors.

miscellaneous_information

## References

[1] Daniel M-C, Astruc D (2004). Chem. Rev.

[2] Sun L, Diaz-Fernandez YA, Gschneidtner TA, Westerlund F, Lara-Avila S, Moth-Poulsen K (2014). Chem. Soc. Rev.

[3a] Maity P, Tsunoyama H, Yamauchi M, Xie S, Tsukuda T (2011). J. Am. Chem. Soc.

[3b] Zhang S, Chandra KL, Gorman CB (2007). J. Am. Chem. Soc.

[3c] Kim Y-H, Gorman CB (2011). Langmuir.

[4a] Chen W, Widawsky JR, Vázquez H, Schneebeli ST, Hybertsen MS, Breslow R, Venkataraman L (2011). J. Am. Chem. Soc.

[4b] Cheng ZL, Skouta R, Vazquez H, Widawsky JR, Schneebeli S, Chen W, Hybertsen MS, Breslow R, Venkataraman L (2011). Nat. Nanotechnol.

[5] Dillon KB, Mathey F, Nixon JF (1998). Phosphorus: The Carbon Copy: From Organophosphorus to Phospha-organic Chemistry.

[6] Woehrle GH, Hutchison JE (2005). Inorg. Chem.

[7a] Weare WW, Reed SM, Warner MG, Hutchison JE (2000). J. Am. Chem. Soc.

[7b] Schmid G, Boese R, Pfeil R, Bandermann F, Mayer S, Calis GHM, van der Velden JWA (1981). Chem. Ber.

[7c] Woehrle GH, Brown LO, Hutchison JE (2005). J. Am. Chem. Soc.

[7d] Pettibone JM, Hudgens JW (2011). ACS Nano.

[8a] Moores A, Goettmann F, Sanchez C, Le Floch P (2004). Chem. Commun.

[8b] Goettmann F, Moores A, Boissière C, Le Floch P, Sanchez C (2005). Small.

[9] Mallissery SK, Gudat D (2010). Dalton Trans.

[10a] Arkhypchuk AI, Mijangos E, Lomoth R, Ott S (2014). Chem. Eur. J.

[10b] Öberg E, Orthaber A, Lescop C, Réau R, Hissler M, Ott S (2014). Chem. Eur. J.

[10c] Orthaber A, Boroucki S, Shen W, Reau R, Lescop C, Pietschnig R (2014). Eur. J. Inorg. Chem.

[10d] Geng X-L, Hu Q, Schäfer B, Ott S (2010). Org. Lett.

[10e] Geng X-L, Ott S (2011). Chem. Eur. J.

[11a] Sugie A, Somete T, Kanie K, Muramatsu A, Mori A (2008). Chem. Commun.

[11b] Wallner A, Jafri SHM, Blom T, Gogoll A, Leifer K, Baumgartner J, Ottosson H (2011). Langmuir.

[11c] Shem PM, Sardar R, Shumaker-Parry JS (2009). Langmuir.

[12] Schäfer B, Öberg E, Kritikos M, Ott S (2008). Angew. Chem. Int. Ed.

[b01] (2008). Angew. Chem.

[14] Orthaber A, Öberg E, Jane RT, Ott S (2012). Z. Anorg. Allg. Chem.

[16a] Freytag M, Ito S, Yoshifuji M (2006). Chem. Asian J.

[16b] Weber L, Lassahn U, Stammler H-G, Neumann B, Karaghiosoff K (2002). Eur. J. Inorg. Chem.

[16c] Ito S, Kusano S, Morita N, Mikami K, Yoshifuji M (2010). J. Organomet. Chem.

[16d] Ito S, Freytag M, Yoshifuji M (2006). Dalton Trans.

[19] Sharma R, Holland GP, Solomon VC, Zimmermann H, Schiffenhaus S, Amin SA, Buttry DA, Yarger JL (2009). J. Phys. Chem. C.

[20a] Link S, El-Sayed MA (1999). J. Phys. Chem. B.

[20b] Li Y, Liu S, Yao T, Sun Z, Jiang Z, Huang Y, Cheng H, Huang Y, Jiang Y, Xie Z, Pan G, Yan W, Wei S (2012). Dalton Trans.

[20c] Schmid G, Alexander BD, Barthelmes J, Mueting AM, Pignolet LH (2007). Inorganic Syntheses.

[20d] Reardon JE, Frey PA (1984). Biochemistry.

