# Supplementary material for: Cooperative Gold Nanoparticle Stabilization by Acetylenic Phosphaalkenes
Source: Angew Chem Int Ed Engl. 2015 Jul 23;54(36):10634–8. doi: 10.1002/anie.201504834 (PMC4557036; doi:10.1002/anie.201504834)

Angewandte Chemie
Eine Zeitschrift der Gesellschaft Deutscher Chemiker

Supporting Information

Cooperative Gold Nanoparticle Stabilization by Acetylenic Phosphaalkenes

Andreas Orthaber, Henrik Löfås, Elisabet Öberg, Anton Grigoriev, Andreas Wallner, S. Hassan M. Jafri, Marie-Pierre Santoni, Rajeev Ahuja, Klaus Leifer, Henrik Ottosson, and Sascha Ott**

anie_201504834_sm_miscellaneous_information.pdf

General Synthesis

All manipulations are carried out under ambient conditions unless otherwise stated. Chemicals are obtained from commercial suppliers and used as received. Solvents are dried over CaH_2 or Na and distilled prior to use. ^{1}H-NMR spectra were recorded on a JEOL Eclipse+ spectrometer operating at a proton frequency of 400 MHz at 25 °C. The spectra were referenced to solvent residual peaks as an internal standard ($CHCl_3$: δ_H = 7.26 ppm) and the measured values for δ are given in ppm. ^{13}C{^{1}H}-NMR and ^{31}P{^{1}H}-NMR measurements were recorded on the same instrument and referenced internally to solvent peaks or externally to $H_3PO_{4(aq.)}$ (85%), respectively.
Phosphaalkenes are prepared according to literature procedures.[1] Gold complex [Au(tht)Cl] was prepared according to literature procedures.[2] All carbon ene-diynes were prepared from their corresponding dibromo-olefins using standard Sonogashira conditions and spectroscopic parameters are identical to reported data.

(Dibromomethylene)(2,4,6-tri-tertbutylphenyl)phosphine gold chloride ([AuCl(**1**)]**)**
Dibromo phosphaalkene **7** (45 mg, 0.1 mmol) and [Au(tht)Cl] 32 mg, 0.1 mmol) was dissolved in 10 ml CH_2Cl_2. The yellow solution was stirred overnight under Ar at r.t. Solvent is removed in vacuo and the residue is washed with water. Slow evaporation of a CH_3Cl solution gave colorless crystals. Yield 54 mg (0.08 mmol, 80%).
^{1}H-NMR ($CDCl_3$, 400 MHz): δ = 7.49 (d, $^4J_{PH}$ = 3.3 Hz, 2 H), 1.56 (s, 18H, ortho-*tert*-Bu), 1.31 ppm (s, 9H, para-*tert*-Bu). ^{13}C-NMR ($CDCl_3$, 100 MHz): due to broadening of the signals no carbon nmr could be recorded. ^{31}P{^{1}H}-NMR ($CDCl_3$, 162 MHz): 233.5 ppm (very broad peak). ESI-MS ($CHCl_3$/MeOH,$AgCF_3$):m/z 1324.97375 (calc. 1324.97204)[M_2-Cl]$^+$, 1093.03760 (cal. 1093.03664) [M_2-($AuCl_2$)].

(E)-(2,4,6-tri-tert-butylphenyl)(4-(trimethylsilyl)but-3-yn-2-ylidene)phosphane ([AuCl(**4**)]**)**
Z-Bromo-,methyl phosphaalkene **4** (64 mg,0.17 mmol) and [Au(tht)Cl] 54 mg, 0.17 mmol) was dissolved in 1015ml CH_2Cl_2. The yellow solution was stirred overnight under Ar at r.t. Solvent is removed in vacuo and the residue is washed with water. The residue is redissolved in CH_2Cl_2 and dried with $MgSO_4$. Removal of all volatiles gives 89 mg of [AuCl(**4**)]. (0.14 mmol, 84%).
^{1}H-NMR ($CDCl_3$, 400 MHz): δ = 7.52 (d, $^4J_{PH}$ = 3.3 Hz, 2 H), 1.60 (s, 18H, ortho-*tert*-Bu), 1.35 (d, $^3J_{PH}$ = 27.0 Hz, Me), 1.33 (s, 9H, para-*tert*-Bu), 0.27 (s, 9H, TMS) ppm. ^{13}C-NMR ($CDCl_3$, 100 MHz): due to broadening of the signals no carbon NMR could be recorded. ^{31}P{^{1}H}-NMR ($CDCl_3$, 162 MHz): 222.3 ppm (broad peak).

(1,5-Bis(trimethylsilyl)penta-1,4-diyn-3-ylidene)(2,4,6-tri-tertbutylphenyl) phosphine gold chloride ([AuCl(**7**)]**)**
Diacetylenic phosphaalkene **1** (65 mg, 0.134 mmol) and [Au(tht)Cl] (43 mg, 0.134 mmol) was dissolved in 20 ml CH_2Cl_2. The yellow solution was stirred overnight under Ar. Solvent removed in vacuo. Recrystallization from CH_2Cl_2/pentane gave light yellow crystals. Yield 92 mg (0.13 mmol, 96%).
^{1}H-NMR ($CDCl_3$, 400 MHz): δ = 7.59-7.58 (br. s overlapping, 2H, Ar), 1.65 (br. s overlapping, 18H, *tert*-Bu), 1.33 ppm (s, 9H, *tert*-Bu), 0.29 ppm (s, 9H, $Si(CH_3)_3$). -0.10 ppm (s, 9H, $Si(CH_3)_3$). ^{13}C-NMR ($CDCl_3$, 100 MHz): 156.2 (Mes*), 154.6 (Mes*), 133.7 (d, J_{PC} = 86.8 Hz), 128.3 (d, J_{PC} = 14.9 Hz), 123.9 (Mes*), 123.8 (Mes*), 122.8 (d, J_{PC} = 25.9 Hz, Mes*), 115.0 (d, J_{PC} = 17.2 Hz, acetylene), 108.9 (d, J_{PC} = 23.1 Hz), 101.3 (acetylene), 100.5 (d, J_{PC} = 21.1 Hz), 39.1, 35.4, 34.1, 31.1, -0.5, -0.8. ^{31}P-{^{1}H}-NMR ($CDCl_3$, 162 MHz): 264.0 ppm EI MS (70 eV): m/z [M^+- AuCl] 482.2 (30), [Mes*P^+-H] 275.3 (100). Elemental analysis (%) calcd. for ($C_{29}H_{47}AuClPSi_2 \cdot CH_2Cl_2$); C 45.03; H, 6.17; Found: C 45.31; H, 6.02.

General Procedure Gold Nanoparticle Synthesis

All glassware and stirring bars are thoroughly clean using a *aqua regia*, rinsed with deionized water and dried at 130 °C for 24 h. Solvents are freshly distilled used immediately.
The ligand (0.03 mmol) and $HAuCl_4$*3(H_2O) are dissolved in 5-10 ml THF. The tetrachloro auric acid is added to the ligand solution and stirred in the dark for the given mixing time. Subsequently, reducing agent (e.g. triethyl silane) is added over a period of 30 sec. and stirred for 1-24 h. The reaction mixture is filtered through normal filter paper and the filtrate is collected. All volatiles can be removed at slightly elevated temperatures (35 °C) under vacuum and the residue is washed with cold methanol. Solutions can also directly be used for NMR investigations or TEM imaging.

Table Experiments

**Entry**	**Ligand**	**Gold source**	**Adds.**	**Reducing agent**	**Ratio**[a]	**Comment**
	Phosphaalkenes					
1	[AuCl(**7**)]<br>Toluene:MeCN (1:4)	-	-	9-BBN	1:0:0:4	No AuNPs
2	[AuCl(**7**)]<br>THF		-	K-naphth.	1:0:0:4	No AuNPs
3	[AuCl(**7**)]<br>THF	$HAuCl_4$	-	Et_3SiH	1:2:0:5	AuNP formation
4	**7**<br>THF	$HAuCl_4$	-	Et_3SiH	1:2:0:50	AuNP formation
5	**7**<br>THF	$HAuCl_4$	PMe_3	Et_3SiH	1:2:1:5	No AuNPs
6	**1**<br>THF	$HAuCl_4$	-	Et_3SiH	1:2:0:5	No AuNPs
7	[AuCl(**1**)]<br>THF	$HAuCl_4$	-	Et_3SiH	1:2:0:5	No AuNPs
8	[AuCl(**1**)]<br>THF	tht*AuCl	-	Et_3SiH	1:2:0:5	No AuNPs
9	**2**<br>THF	$HAuCl_4$		Et_3SiH	1:2:0:50	No AuNPs
10	**3**<br>THF	$HAuCl_4$		Et_3SiH	1:2:0:50	No AuNPs
11	**4**<br>THF	$HAuCl_4$		Et_3SiH	1:2:0:50	AuNP formation
12	[AuCl(**4**)]<br>THF	$HAuCl_4$		Et_3SiH	1:2:0:50	AuNP formation
13	**5**<br>THF	$HAuCl_4$		Et_3SiH	1:2:0:50	AuNP formation
14	**6**<br>THF	$HAuCl_4$		Et_3SiH	1:2:0:50	AuNP formation
	All carbon analogs					
15	**8**<br>THF	$HAuCl_4$		Et_3SiH	1:2:0:5	No AuNPs
16	**9**<br>THF	$HAuCl_4$		Et_3SiH	1:2:0:5	No AuNPs
17	**10**<br>THF	$HAuCl_4$		Et_3SiH	1:2:0:5	No AuNPs
18	**11**<br>THF	$HAuCl_4$		Et_3SiH	1:2:0:5	No AuNPs

[a] Ratio of Ligand:gold:additive:reducing agent

X-ray crystallography.

Crystallographic data sets were collected from single crystal samples mounted on a loop fiber and coated with N-paratone oil (Hampton Research). The collection was performed using a Bruker SMART APEX diffractometer equipped with an APEXII CCD detector, a graphite monochromator and a 3-circles goniometer. The crystal-to-detector distance was 5.0 cm, and the data collection was carried out in 512 x 512 pixel mode. Cell refinement and data reduction were performed with SAINT V7.68A (Bruker AXS) on the final data. Absorption correction was done by multi-scan methods using SADABS96 (Sheldrick). The structure was solved by direct methods and refined using SHELXL97. All non-H atoms were refined by full-matrix least-squares with anisotropic displacement parameters while hydrogen atoms were placed in idealized positions. Refinement of F^2 was performed against all reflections. The weighted R-factor wR and goodness of fit S are based on F^2. Crystal structures data were deposited at the Cambridge Crystallographic Data Centre and can be retrieved free of charge quoting deposition numbers 809644 and 1033018.

Compound	[AuCl(**1**)]	[AuCl(**7**)]
Empirical formula	$C_{29}H_{47}AuClPSi_2,(C_3H_7)_{0.5}$	$C_{38}H_{58}Au_2Br_4Cl_2P_2$
Formula weight [g mol^{-1}]	736.78	1361.26
CCDC-No.	809644.	1033018
Crystal system	Monoclinic	Monoclinic
Space group	C2/c	$P2_1/n$
Unit cell dimensions a[Å]	19.021(11)	14.3526(7)
b [Å]	19.628(10)	10.5589(5)
c [Å]	20.340(12)	15.8833(8)
α, β, γ [°]	90, 104.031(11), 90	90, 107.2690(10), 90
Volume [Å^3], Z	7368(7), 8	2298.57(19), 2
Density [g cm^{-3}]	1.328	1.967
μ(Mo$K_α$) [mm^{-1}]	4.190	10.063
F(000)	2980	1296
Crystal size [mm]	0.25 x 0.20 x 0.13	0.25 x 0.22 x 0.12
Crystal Description	colorless plate	colorless block
Temperature [K]	100(2)	100(2)
Radiation [Å]	0.71073	0.71073
Θ range min, max [°], completeness	1.51 to 31.22<br>0.834	1.68 to 31.21,<br>0.952
Index ranges	-27 ≤ h ≤ 24, -24 ≤ k ≤ 16,<br>-20 ≤ l ≤ 29	-20 ≤ h ≤ 18, -15 ≤ k ≤ 15,<br>-23 ≤ l ≤ 22
Reflections collected	69275	43301
Reflections unique	9984	7099
Parameters, restraints	327, 0	228, 0
R(int)	0.085	0.0196
Gof (F^2)	0.916	1.018
R_1 [I > 2σ(I)]	0.0458	0.0210
wR_2 (all data)	0.1259	0.0491
larges difference peak/hole [e A^{-3}]	1.979 / -2.503	0.832 / -1.153

Raman spectroscopy:

Measurements were performed on a performed on a Renishaw micro-Raman spectrometer (Modell 1000) equipped with a 514 nm Ar laser and a 1200 lines/mm grating. The spectra are reference with a Si-reference sample. The free ligands were recorded as solid samples on an amorphous glass substrate with exposure times of 10 seconds, a laser power of 25% and accumulation of 2 scans. Longer exposure times, more scans or higher laser power were precluded due to sample decomposition. The spectra were baseline corrected with a 5th order polynomial. The AuNPs are measured as solid samples after extensive purification as described in the synthetic procedures. In total 3 accumulated scans à 10 seconds with a laser power of 100% compose the final spectra. The spectra were processed with the WIRE2 smooth (version 2.1.0.13) and baseline subtraction (version 2.1.0.12) suite of programs.

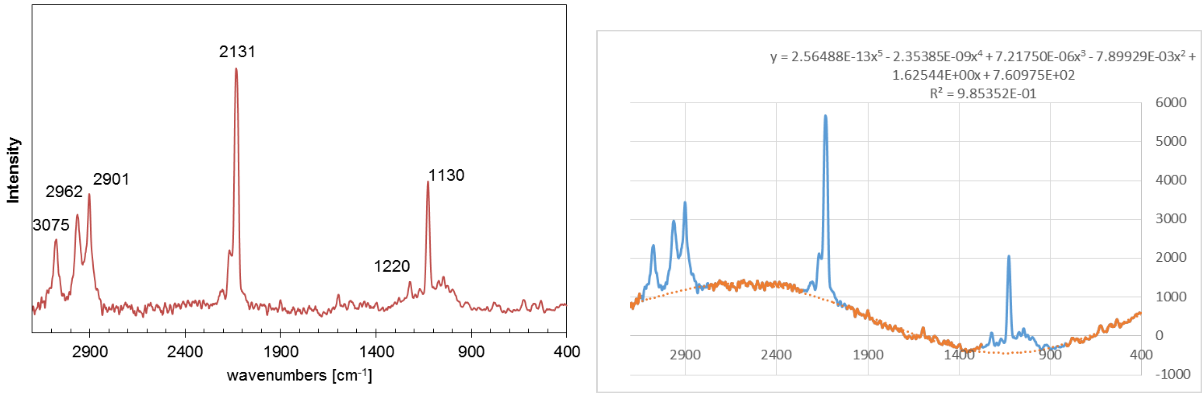

Raman spectra of solid free ligand **4** (left, fully processed). Due to the low intensity of the signal and poor S/N ratio an extended base line correction was needed (right, details of base line correction)

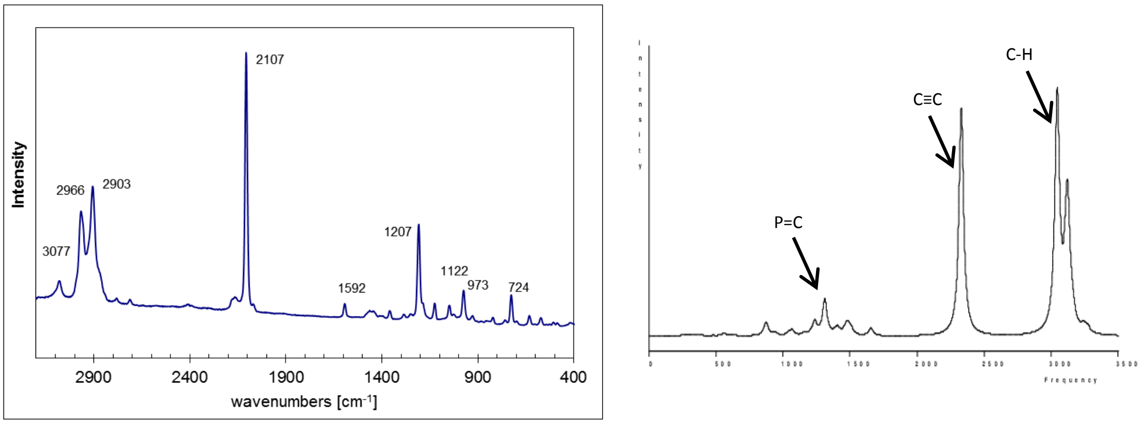

Surface enhanced Raman spectra of phosphaalkene **4** decorated AuNPs (left). Calculated Raman spectra of ligand 4 at the PBE1PBE/ 6-311G** density functional level of theory (plotted and analyzed with MOLDEN).

Computational methods.

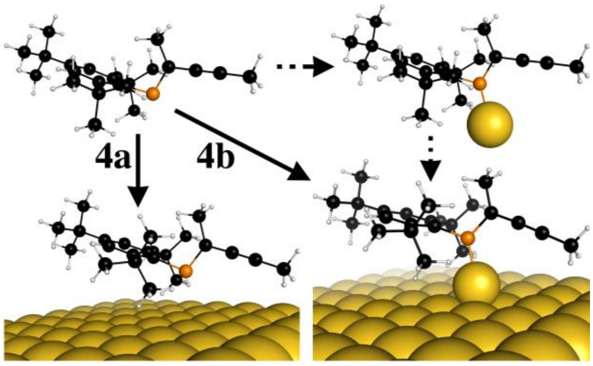

Figure 1. Adsorption energies calculation for compounds **4** (left) and **4** + adatom (right).

The relaxed molecular structures were inserted on top of Au(111)-surface and relaxed with 4 gold layers beneath. To remove interactions between periodic images in the lateral dimensions, a 8×8 supercell (monoclinic with a=b=23.59 Å) was used and more than 17 vacuum layers were introduced to accommodate the molecule (c=51.68 Å). Two layers of gold are fixed on the other side of the slab to model their bulk positions. All relaxations are performed at the DFT-level with the SIESTA package[3,4] and core electrons are modeled using Troullier-Martins[4] soft norm-conserving pseudo-potentials. The valence electrons are expanded in a basis set of local orbitals using a double-zeta plus polarization orbitals (DZP) basis set for electrons in the molecule and a single-zeta plus polarization orbitals (SZP) basis set for electrons in the gold particle. The GGA of Perdew-Burke-Ernzerhof was used for the exchange-correlation functional.[6] The reciprocal cell was sampled with a 5×5×3 k-mesh. We have additionally considered a more densely packed film of molecules either in a laterally smaller cell or with two molecules per cell, but the additional stabilization of the system by intermolecular interactions is quite similar for all molecules and does not explain experimentally observed behavior for different compounds. In these calculations we heave also checked convergence of our calculations with respect to the slab thickness and basis size (thicker gold slab and DZP basis for surface gold atoms were tested) and found it satisfactory.

Here we started by modeling the AuNPs by a flat gold (111)-surface resulting in a Au-P bond distance of more than 4 Å for the different calculations indicating a very weakly bond system. Adsorption energies are slightly negative indicating that the systems are not adsorbed at all, but theses systems should be weakly physisorbed and the dispersive forces are not fully captured in our approach.

The AuNPs can be expected to contain a number of defect sites of the perfect Au(111) surfaces. To model these defects, we consider a gold ad atom on the previous used surface. In these cases it is possible for the phosphorus lone pairs to coordinate with the defect gold atom, forming a stronger bond. We calculate an Au-P bond distance of about 2.35 Å, more in agreement with a chemisorbed (coordinated) molecule. In agreement, we also get adsorption energies of about 1 eV.

To elucidate the difference in the adsorption mechanism, we study Crystal Orbital Hamilton population (COHP). COHP is a way to quantify the density of states (PDOS) from the DFT-calculations into bonding, anti bonding and non-bonding states, and opens the possibility to

calculate the bond strength.[7] By integrating the COHP-curves up to the Fermi level (E_F) it is possible to obtain the bond energy for the given interaction (or actually the change to the band-structure energy due to this interaction). Positive peaks in the COHP corresponds to anti-bonding and negative to bonding interactions.[3,4]

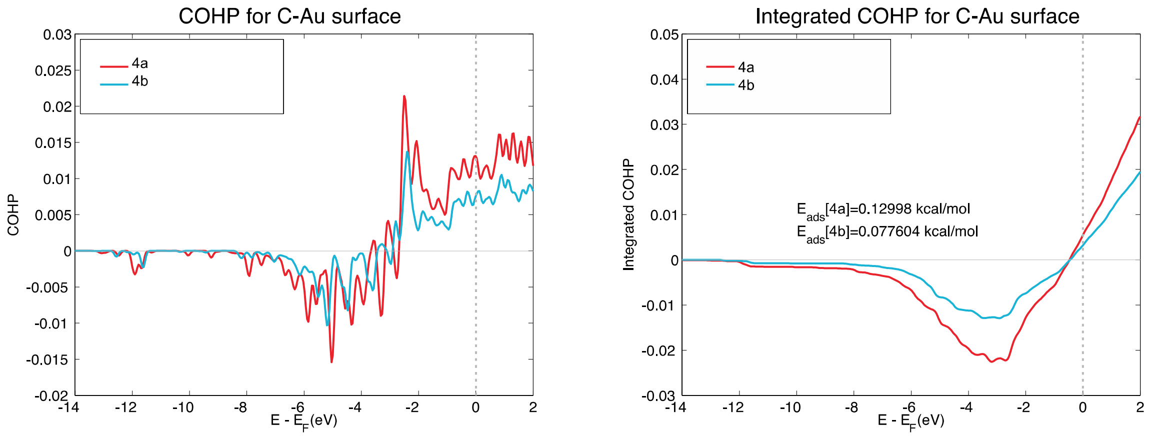

Figure 2: Summed up COHP (left panel) for all C atoms in the molecules and the underlying Au-surface (not including the adatom). The right panel shows the integrated COHP.

For example, we consider the total interaction between all the carbon atoms (excluding the P atom) in the molecules and the underlying flat gold surface (excluding the ad-atom), as shown on Fig. 2. the carbon system is not bonding to the gold surfaces (positive value of the integrated COHP at E_F)

The carbon atoms closest to P interact very similar with Au ad-atom yielding (yielding E_{ads}[P=CMe]= 0.1325 kcal/mol), the atoms participating of the triple bond bind to the adatom with E_{ads}[C1]=−0.069705 kcal/mol and E_{ads}[C2]=−0.013073 kcal/mol. As one can see, this binding is comparable in strength to non-bonding interaction of all carbons in the molecule to underlying flat surface (not counting the ad-atom).

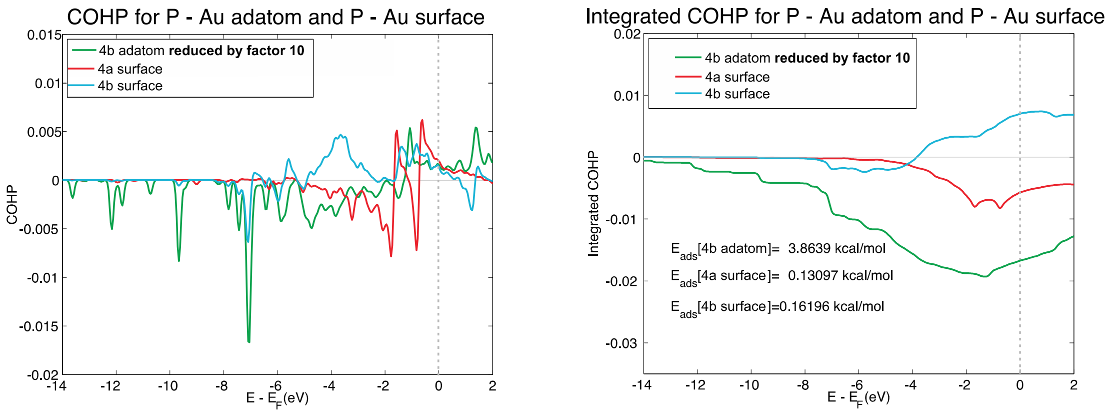

Figure 3: Summed up COHP (left panel) for the P atom and the underlying Au-surface (not including the ad-atom) and the Au ad-atom (latter is reduced by factor 10). The right panel shows the integrated COHP.

Interaction between the P atom and the underlying gold is more complicated, as shown on Fig. 3. Here the P-atom's interaction is bonding to the underlying flat gold surface. Adding the extra gold makes phosphor interaction anti-bonding to the bulk gold.

The interaction between the P atom and the extra defect on gold - ad-atom demonstrate a significantly stronger interaction (factor 10 scaling is applied to fit into the same graph) of about -3.86 kcal/mol.

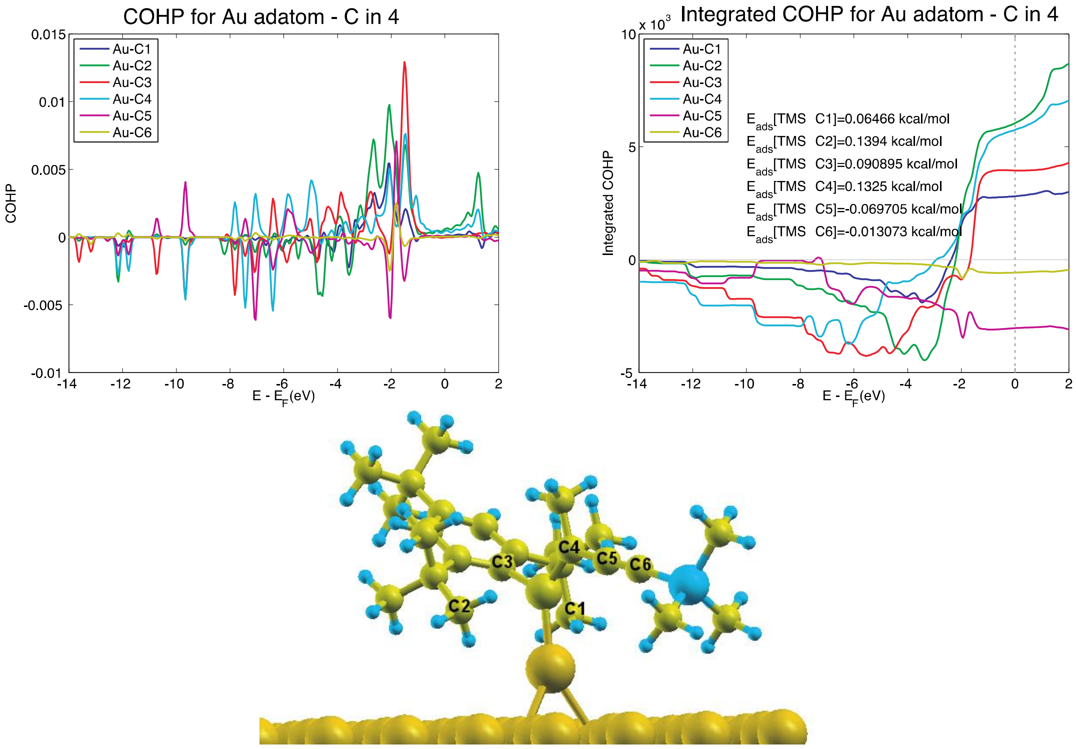

Fig. 4: Crystal Orbital Hamilton population (COHP) - the projected density of states (PDOS) from the DFT-calculations weighted with bond strength: bonding (negative) and antibonding (positive) of the different closest C-atoms marked C1 to C6 in 4.[3,4] Note the two states above -2 eV localized at the acetylenic bond which have pronounced bonding character. As evident from the negative values of the integrated CHOP at the Fermi level for C5 and C6 we deduce an additional stabilization of the ligand through the acetylenic unit.

References

1. (a) Schäfer, B.; Öberg, E.; Kritikos, M.; Ott, S., Incorporating Phosphaalkenes into Oligoacetylenes. *Angew. Chem., Int. Ed. Engl.* **2008,** *47* (43), 8228-8231; (b) Öberg, E.; Schäfer, B.; Geng, X.-L.; Pettersson, J.; Hu, Q.; Kritikos, M.; Rasmussen, T.; Ott, S., C,C-Diacetylenic Phosphaalkenes as Heavy Diethynylethene Analogues. *J. Org. Chem.* **2009,** *74* (24), 9265-9273; (c) Orthaber, A.; Öberg, E.; Jane, R. T.; Ott, S., Alternative synthesis and structures of C-monoacetylenic phosphaalkenes. *Z. Anorg. Allg. Chem.* **2012,** *638* (14), 2219–2224.

2. Uson, R.; Laguna, A.; Laguna, M.; Briggs, D. A.; Murray, H. H.; Fackler, J. P., (Tetrahydrothiophene)Gold(I) or Gold(III) Complexes. In *Inorg. Synth.*, John Wiley & Sons, Inc.: 2007; pp 85-91.

3. Dronskowski, R.; Bloechl, P. E., Crystal orbital Hamilton populations (COHP): energy-resolved visualization of chemical bonding in solids based on density-functional calculations. *J. Phys. Chem.* **1993,** *97* (33), 8617-8624.

4. http://www.cohp.de/

Representative NMR and UV data of selected samples:

Entry 4: compound 7, $HAuCl_4$, Et_3SiH 1:2:50

NMR of the crude reaction mixture shows the formation of new species which we tentatively assigned to the protodesilylalted phosphaalkenes

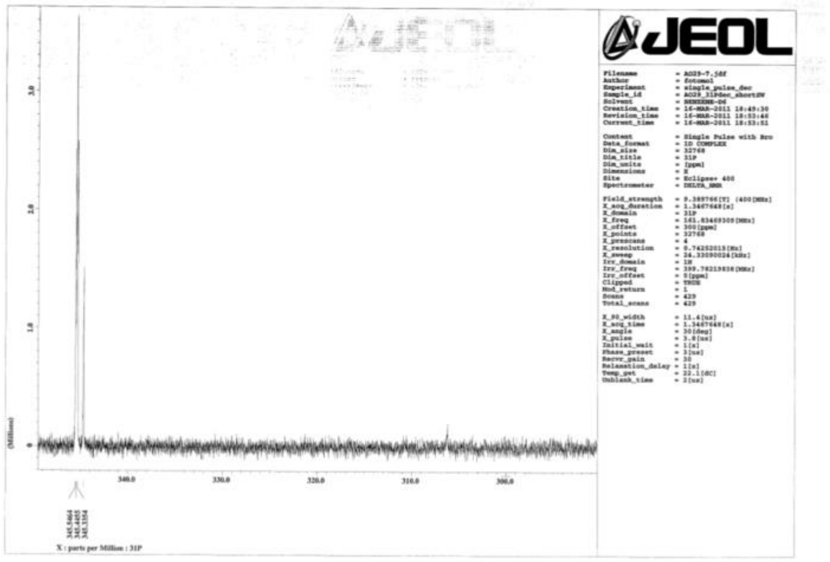

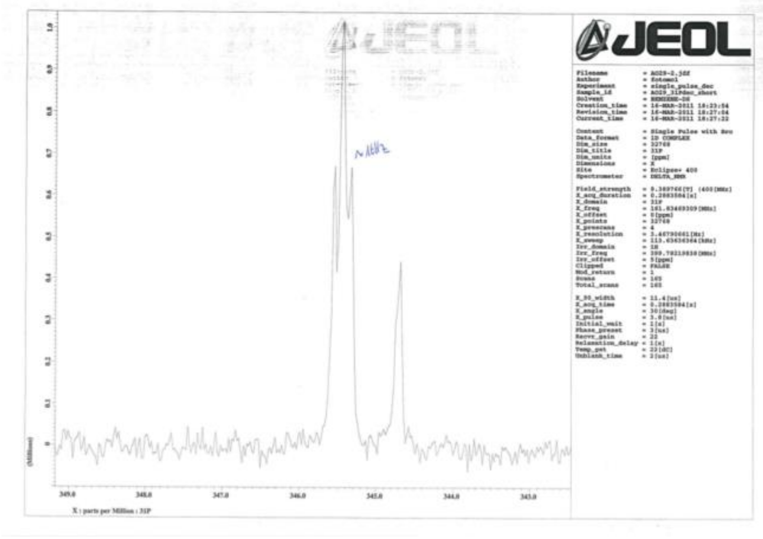

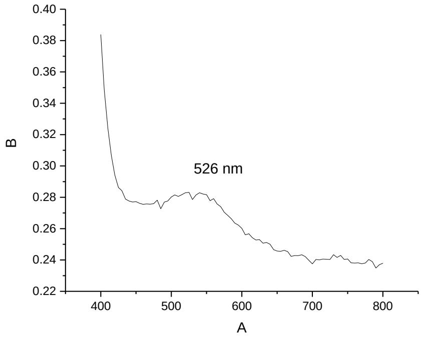

UV/vis dataof the crude AuNP solutions indicating the presence of the typical plasmonic resonance around 520 nm indicative for the formation of AuNPs larger than 1.5- 2 nm.

Entry 11: Compound 4, $HAuCl_4$, Et_3SiH, 1:2:0:50.

Comparison of purified and crude AuNPs. The crude AuNPs solutions were gently dried vacuum and redissolved in C_6D_6. Purification of the AuNPs after removal of all volatiles and subsequent extensive washing cold methanol. The residue was redissolved in C_6D_6.

In the crude samples the ^{31}P and ^{1}H-NMR data suggest he presence of intact phosphaalkene species in solution, i.e. not bound to the gold surface. After purification almost all dissolved ligands were removed, however no additional species were detected that could be assigned to surface bound phosphaalkenes.

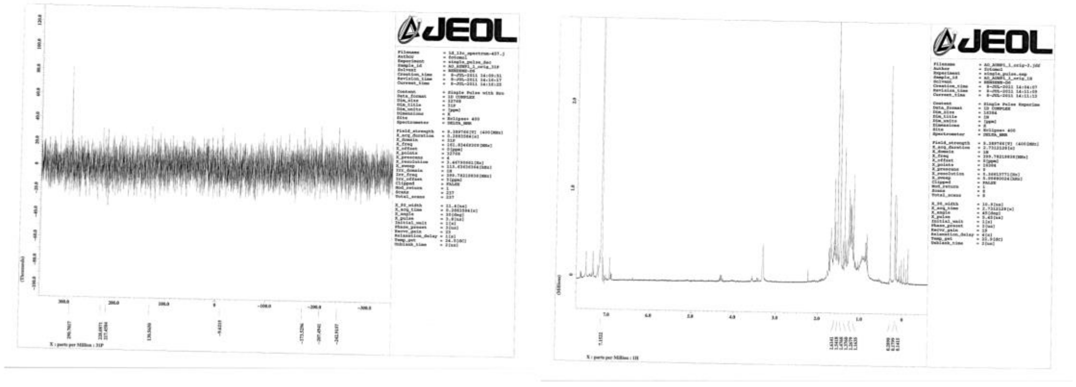

Crude Nanoparticle solution

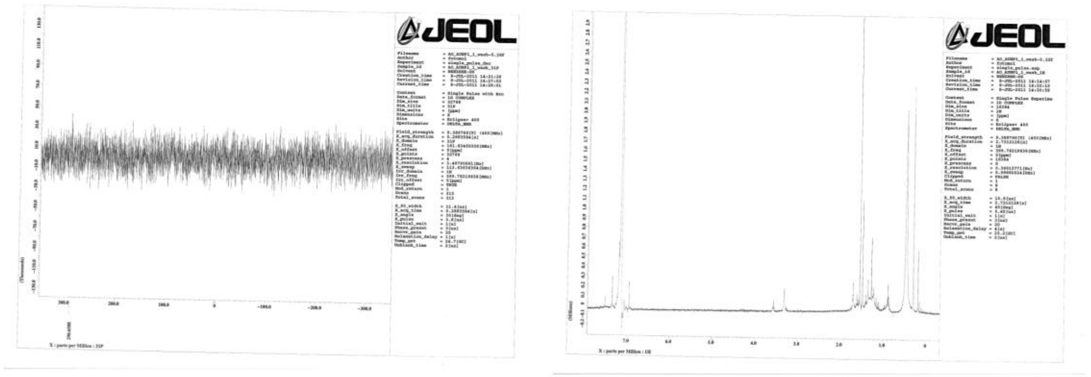

Nanoparticle solution after purification

UV/data of AuNPs before and after purification indicating no changes of the AuNPs during the purification procedure

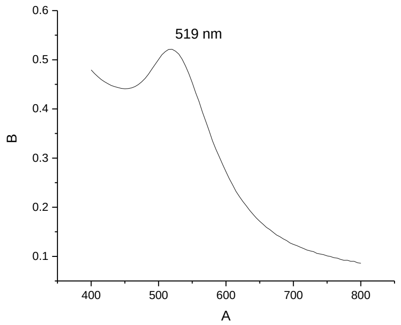

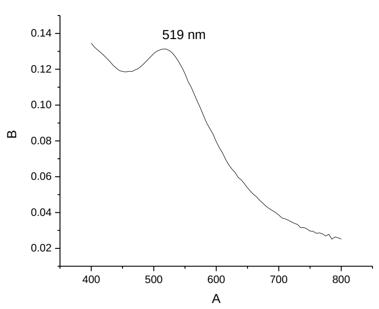

NMR data for the previsouly non reported gold complexes and [AuCl*1]. The ^{31}P-NMR resonance shifts to higher fields as compared to the uncomplexed ligand (i.e. δ^{31}P (**1**) = 270 ppm) typical for π-backbonding metals. For the broadening of the ^{31}P signal we could not find a rational explanation.

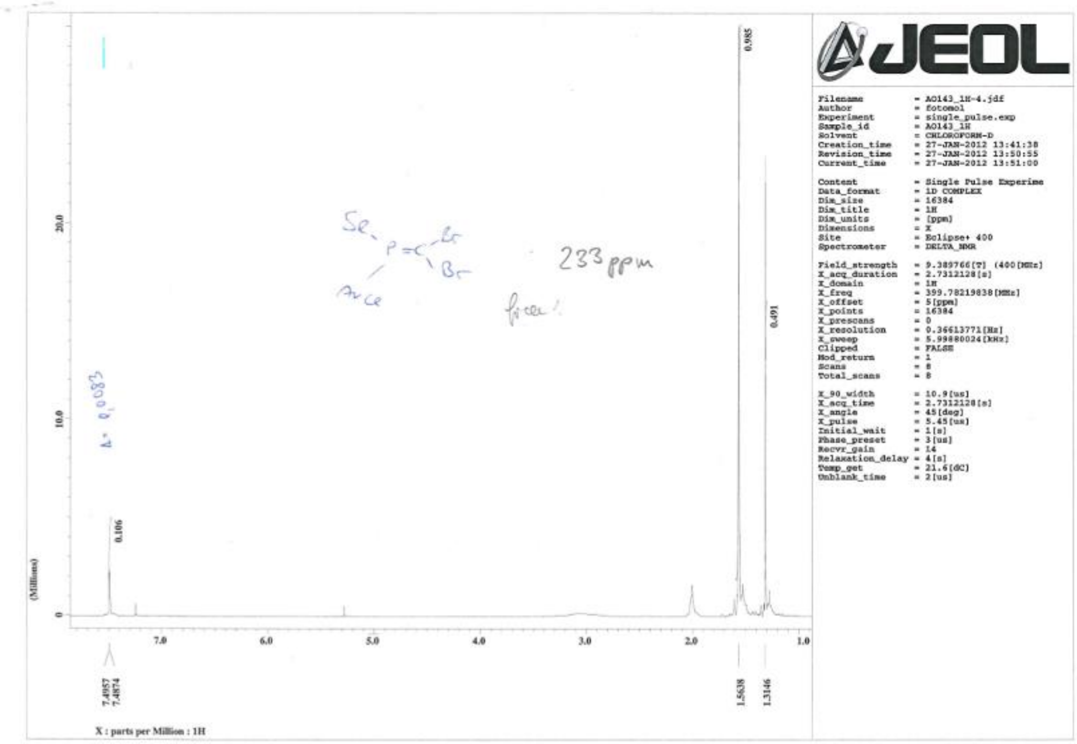

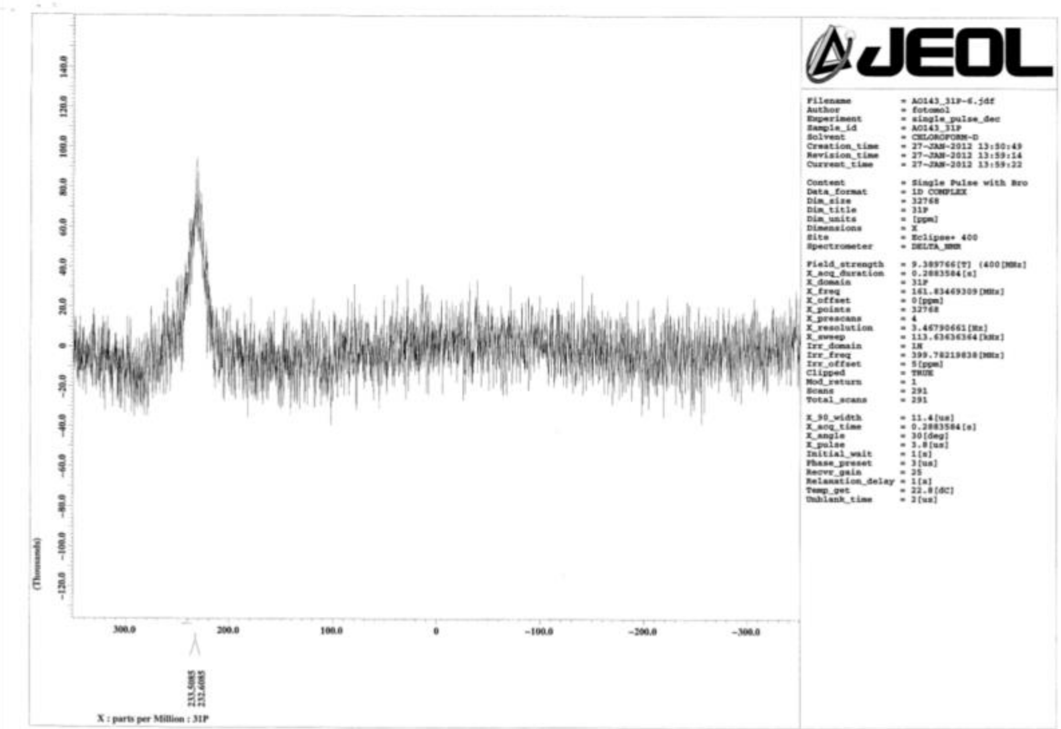

NMR data of previously non-reported gold complex [AuCl***4**]: ^{13}C-NMR data could not be recorded due to broadening of the lines, which is in line with the low intensity and the significant broadening of the resonance observed in ^{31}P-NMR.

Compound [AuCl*4]

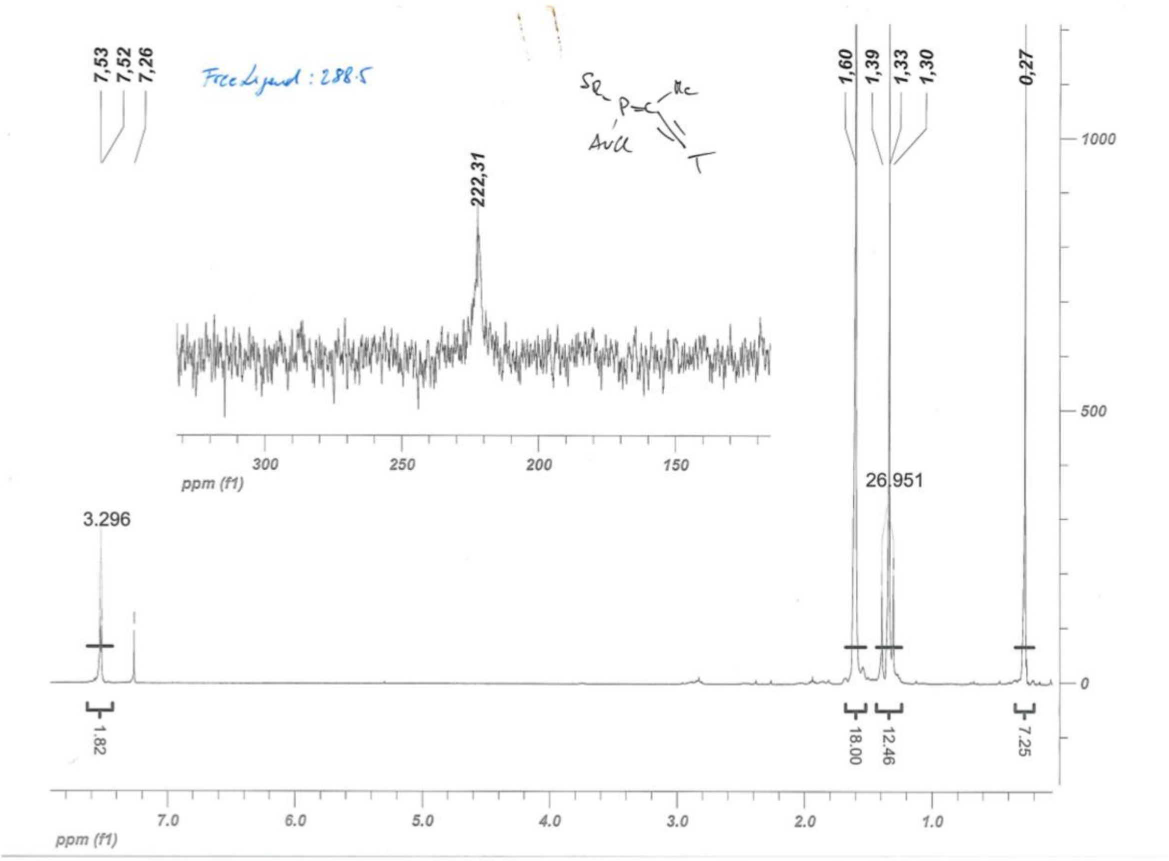

Supplement: Supplementary file 1 — miscellaneous_information [file anie0054-10634-sd1.pdf]
